# The Validity of Motion Capture Analysis System against the Gold Standard Long-Standing Radiography in the Measurement of Lower Extremity Alignment

**DOI:** 10.3390/jcm12020567

**Published:** 2023-01-10

**Authors:** Robert Ossendorff, Johanna Richter, Etienne Heijens, Frank A. Schildberg, Gordon Haeder, Gian M. Salzmann

**Affiliations:** 1 Department for Orthopedics and Trauma Surgery, University Hospital Bonn, 53127 Bonn, Germany; 2 Institute of Sports Science, Johannes Gutenberg University Mainz, 55128 Mainz, Germany; 3 Gelenkzentrum Rhein-Main, 65239 Hochheim, Germany; 4 Schulthess Clinic, 8008 Zurich, Switzerland

**Keywords:** motion captured analysis, malalignment, long-standing full leg radiographs, X-ray

## Abstract

Motion capture analysis (MCA) has the advantage of providing a static and dynamic leg axis analysis without radiation. Nevertheless, there is a lack of evidence regarding the accuracy of this technique. To test whether mechanical femorotibial axis angle (MAA) measurement recorded with a non-invasive MCA system is equal to the gold standard static long-standing full-leg radiographs (LSX) and if the degree of malalignment or other parameters (BMI, body mass, height, age) influence the accuracy, a total of 102 consecutive patients were examined using LSX and MCA. Static as well as all gait motion phases at 3 km/h were analyzed regarding the difference between the two angles. There was no statistical difference for MAA between LSX (MAA_rad_) and MCA (MAA_stat_) (*p* = 0.091). There was a strong correlation (r_s_ = 0.858, *p* < 0.001) between the two methods. The highest accuracy was detected for values of standing MCA. Also, the gait MCA values showed strong correlation with LSX but weaker correlation compared to standing MCA (initial swing r_s_ = 0.549; terminal stance r_s_ = 0.815; *p* < 0.001). BMI, body mass, and height did not influence the accuracy of MCA. MCA enables frontal alignment analysis with high accuracy and without the side effect of radiation.

## 1. Introduction

### 1.1. Background

Malalignment of the knee joint is often associated with early degeneration. Epidemiologic data from the Multicenter Osteoarthritis Study (MOST) revealed that leg malalignment is often associated with non-physiological loading of the knee joint, resulting in symptomatic cartilage lesions, pain, and decreased function [1]. Moreover, varus deformity is associated with an increased risk of developing osteoarthritis (OA) [1]. If OA has occurred, valgus and varus malalignment increases the risk of further knee OA progression [1,2]. For successful regenerative therapy, a static and dynamic evaluation of malalignment has to be addressed in a therapeutic concept [3]. If leg axis is not corrected, there is a high risk of treatment failure, especially in cartilage repair, but leg axis also has to be considered in ligament surgery.

### 1.2. Rationale

The current gold standard of leg axis evaluation is a standing full lower extremity static radiograph to determine the anatomic and mechanical leg axis [4]. Nevertheless, an exposition to ionizing radiation with the cumulative and stochastic risk of developing cancer must be considered using radiographs [5]. Furthermore, there are alignment discrepancies when directly comparing standing/passive to dynamic gait situations. Such remain undetected during static long-standing full leg radiographs (LSX) and must be quantified via clinical examination and description. Alternative strategies have been reported for measuring leg axis alignment, including physical examinations with goniometers and inclinometers and analysis of the inter-condylar and inter-malleolar distances, but they show significantly lower accuracy in comparison to the gold standard lower extremity X-ray [6,7,8]. Motion capture is a non-invasive technique for measuring leg alignment and enables static and dynamic evaluation using reflective markers on bony landmarks on the skin [9]. Currently, there is a lack of evidence regarding the accuracy of this technique. The aim of this study was to (1) evaluate the accuracy of marker-based motion capture measurement compared to the gold standard full leg X-ray and (2) to analyze the influence of the degree of malalignment, BMI, height, grade of osteoarthritis, and body mass in an adult cohort of an orthopedic outpatient clinic.

## 2. Materials and Methods

### 2.1. Ethics and Patient Cohort

The presented study was approved by the local ethics committee with the ID: 2020-2061-evBO. A total of 102 consecutive subjects were included in the study. The patients presented to the outpatient clinic demonstrated knee joint pain. All patients underwent a complete clinical examination prior to LSX and motion captured analysis (MCA). Patients with an indication of a LSX for at least one knee to analyze clinical valgus/varus deformity were included in the study. MCA was performed as an additional routine diagnostic in this cohort. Results of both methods, LSX and MCA, were performed in the outpatient clinic. Exclusion criteria were an extension deficit of more than three degrees and advanced osteoarthritis with a Kellgren and Lawrence score of ≥2 to select a homogenous study population. Knee extension was measured with a goniometer. Patients who were not able to walk on a treadmill were excluded. A total of 105 patients fulfilled the inclusion criteria. Three patients were excluded due to missing data. Body mass index evaluation was divided according to the World Health Organization (WHO) criteria into five categories (underweight: BMI < 18.5; normal weight: 18.5–24.9; pre-obesity: 25–29.9; obesity class 1: 30–34.9; obesity class 2: 35–39.9; obesity class 3: ≥40). BMI, body mass, height, age, and osteoarthritis score were recorded from the patient chart.

### 2.2. Radiographic and Motion Capture Analysis

**Figure 1:**
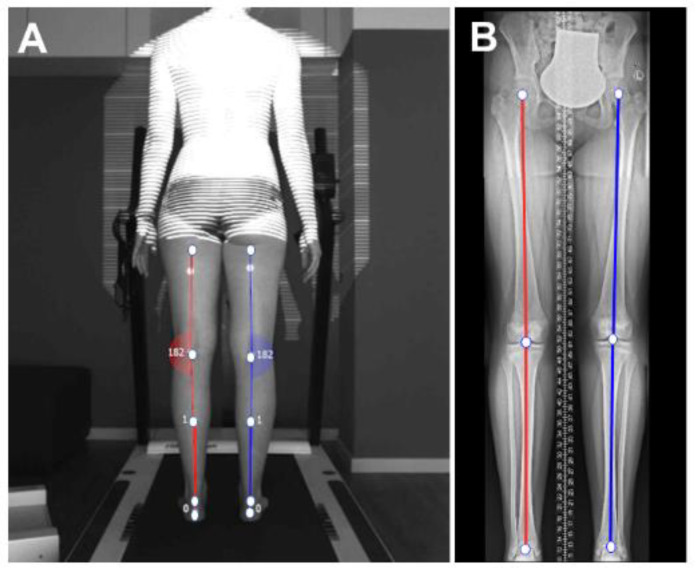
Representation of (**A**) Motion capture analyses (MCA): non-invasive 2D motion capture leg axis evaluation (Diers leg axis posterior^®^). Markers are placed on five anatomical landmarks of the skin (gluteal rim, middle of the popliteal fossa, middle underneath the bellies of the musculus gastrocnemius, calcaneus top edge and calcaneus lower edge) and leg axis analysis is performed; and (**B**) long-standing full-leg radiograph (LSX): mechanical axis angle (MAA_rad_) is defined by the line from the hip center to the knee center and the line from the knee center to the ankle center.

For radiographic alignment analysis, patients were barefoot with knees fully extended in the forward foot position with the patella centered to avoid foot rotation. Radiographic analysis was performed with a full-length standing anteroposterior radiograph (Carestream Ascend DRX, Stuttgart, Germany) (Figure 1). Radiographic MAA (MAA_rad_) was calculated by two independent observers blinded to the marker-based results with a commercially designed evaluation software (STARC medical^®^, Isernhagen, Germany). The average of both calculations was used. The MAA_rad_ corresponded to the angle formed by the line from the hip center to the knee center (inter-condylar midpoint) and the line from the knee center to the ankle center (inter-malleoli midpoint) [4]. Neutral alignment was defined as 180°, varus malalignment as >180°, and valgus malalignment as <180°. Also, the Kellgren and Lawrence score was quantified for all knee joints [10].

A camera-based posture and gait analysis system (DIERS leg axis, DIERS International GmbH, Schlangenbad, Germany) was used to assess the MAA in the coronal plane from the posterior direction [11]. The system orientation requires a centered and orthogonal position of the recording unit behind a treadmill (Valiant 2 rehab, Lode B.V., Groningen, The Netherlands). The camera (UI-3240 CP-M-GL Rev.2, IDS Imaging, Obersulm, Germany) provided a 60 Hz sample rate for an exposure of 8 ms and was equipped with an optical 8-mm lens (Edmund TECHSPEC UC-Series, 8 mm ½”, Edmund Optics GmbH, Mainz, Germany). Data recording, processing, and export were performed with a system-exclusive software (DICAM 3, DIERS International GmbH, Schlangenbad, Germany).

Passive reflective markers (3M, Saint Paul, MN, USA: 5-mm radius, flat, adhesive) were placed on 5 anatomical landmarks on the skin (gluteal rim, middle of popliteal fossa [pf], intersection of m. gastroc. bellies, and top and lower edge of calcaneus). During data acquisition, markers were illuminated with red LED light, thereby creating contrasting local areas on the skin surface, allowing for an automated detection with a left–right/top-to-bottom logic during automated data processing. Markers acted as dividers of the leg into smaller rigid segments, building up a plane-bound orientation of those, identifiable as the mechanical axis [12]. Accordingly, the knee angle derived from connecting the lines between the gluteal rim and [pf] and [pf] to the calcaneus top edge as two angle arms, creating a vertex at [pf], which built up a neutral MAA in vertical alignment with the angle arms at 180° [13]. A coronal vertex deviation represented a valgus (to medial, <180°) or varus (to lateral, >180°) tendency, expressed by the difference between 180° and the angle actually found.

Measurement was performed in static (MAA_stat_) and dynamic conditions. In the dynamic evaluation, gait speed on a treadmill was 3 km/h and the MAA was measured with one single frame in the 8 different phases of the gait cycle, according to Perry et al. [14] (MAApsw: pre swing, MAAisw: initial swing, MAAmsw: mid swing, MAAtsw: terminal swing, MAAic: initial contact, MAAlr: loading response, MAAms: mid-stance, MAAts: terminal-stance).

### 2.3. Statistics

Statistical data analysis was performed with SPSS (version 27, IBM Corporation, Armonk, NY, USA). The Kolmogorov–Smirnov test confirmed non-normal distribution of the analyzed parameters. The Wilcoxon signed-rank test was used to test paired differences in MAA between LSX and MCA. The non-parametric Spearman correlation coefficient (r_s_) identified correlations between radiographic and marker-based measurements and the association of the BMI, body mass, height, age, and OA level. Correlations of r_s_ below 0.3 were defined as low, 0.3 to 0.65 as medium, and above 0.65 as high. ANOVA was used to evaluate the regression coefficient. The significant level was defined as *p* ≤ 0.05. The Bland–Altman plot was used to directly compare and visualize the tested measurement techniques. Agreement between both methods and the potential bias were calculated and visualized.

## 3. Results

### 3.1. Descriptive Results of the Cohort

One hundred and two patients with an age between 16 and 85 years (mean 53.8 ± 15.4 years) were included. A total of 133 data sets were available. Among the cohort, there were measurement data for both LSX and MCA for 69 left and 64 right legs. The difference between patients and data sets was caused by the analysis of both legs in 32 patients (Table 1). Seventy-one percent of the patients had a genu varum (MAA_rad_ > 180°) and 29% had a genu valgum (MAA_rad_ < 180°). Among the cohort, 12.7% suffered from an acute injury, 34.7% had acute pain without injury, and 52.6% had a chronic disease. Among the cohort, average in body mass was 85.3 kg (±18.7 kg). Body mass index was at mean 27.2 (±4.6) kg/m^2^, which is in line with epidemiological data of the German population. A total of 32.4% had normal body mass and the majority (43.1%) were classified as pre-obese. Obesity was detected in 20.5%. The Kellgren–Lawrence score was either 0 or 1 among all subjects and did not affect any values of this outcome analysis.

### 3.2. Comparison of the Agreement between Long-Standing Radiography and Motion Capture Analysis in Alignment Analysis (MAA)

**Figure 2:**
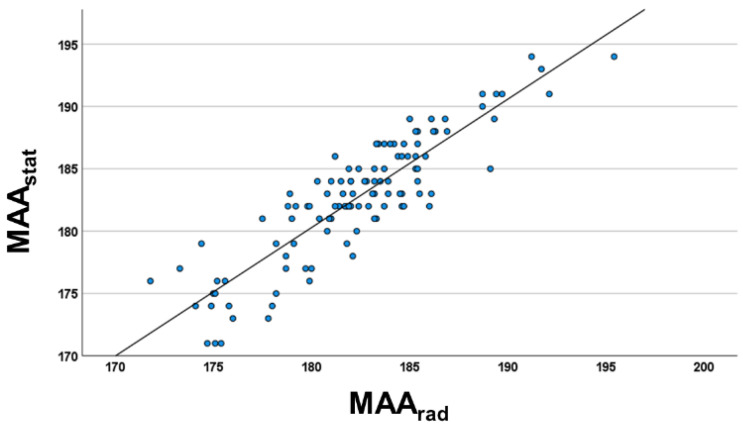
Scatter diagram of the relationship between radiographic femorotibial mechanical axis in frontal plane (MAA_rad_) and marker-based static full leg analysis (MAA_stat_) (r_s_ = 0.858; *p* < 0.001) of the study cohort (*n* = 102). Regression equation: MAA_rad_ = 1.03 MAA_stat_—5.38.

The radiographic MAA (MAA_rad_) of the cohort was between 171.8° (valgus) and 195.4° (varus). There was no statistical difference between MAA_rad_ and MAA_stat_ values (*p* = 0.091). Marker-based 2D MAA_stat_ was highly correlated with MAA_rad_ (Figure 2; r_s_ = 0.858, *p* < 0.001). The comparison analysis between MAA_rad_ and dynamic angles revealed that no statistical difference was detected in the Wilcoxon test for mid-stance, terminal-stance, and pre-swing gait phases. Nevertheless, the correlation coefficient (r_s_) was weaker compared to MAA_stat_. The strongest correlation of dynamic MAA values was detected in mid-stance (r_s_ = 0.815, *p* < 0.001), the weakest correlation in initial swing (r_s_ = 0.549, *p* < 0.001). There were no statistical differences between the results in varus and valgus malalignment.

**Figure 3:**
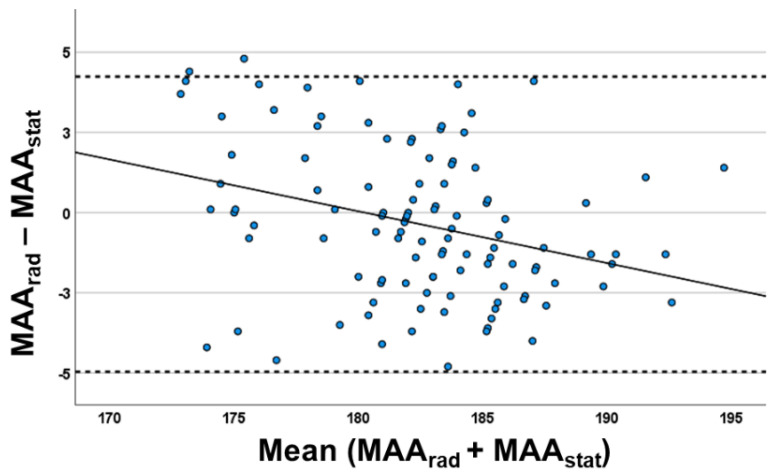
Bland–Altman plot between static marker-based (MAA_stat_) and radiographic leg axis (MAA_rad_) and evaluation (*n* = 102) with: *X*-axis valgus alignment (<180°) and varus alignment (>180°); *Y*-axis: according to the MAA_rad_–MAA_stat_ calculations, the positive values represent the cases for which MAA_rad_ values were greater than those calculated with MAA_stat_ and the negative values represent the cases for which MAA_stat_ values were greater than those calculated with MAA_rad_. The systematic bias of both methods is 2.35°.

**Figure 4:**
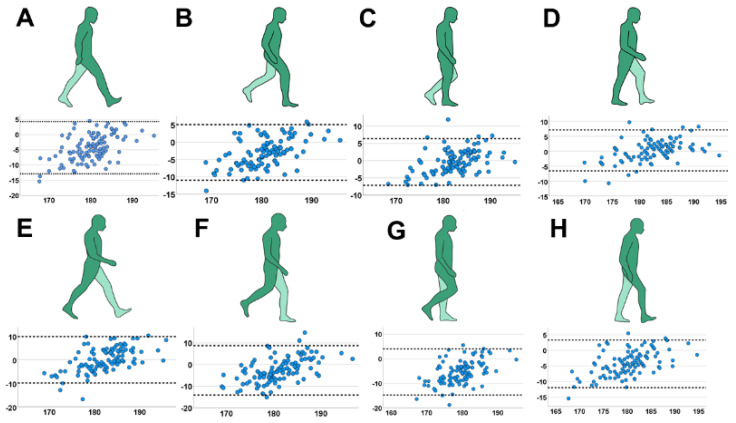
Leg axis analysis in specific gait cycles with Bland–Altman plot indicating systemic bias of the MAA (*X*-axis: mean MAA between static radiograph and marker-based measurement; *Y*-axis: difference in MAA between both techniques). (**A**) initial contact; (**B**) loading response; (**C**) mid- stance; (**D**) terminal-stance; (**E**) pre-swing; (**F**) initial-swing; (**G**) mid-swing; (**H**) terminal-swing.

The Bland–Altman plot analysis of the marker-based static leg axis analysis vs. X-ray showed (Figure 3) a distribution of almost all measurements inside the range (defined as ±1.96 × standard deviation + mean), which indicates a good comparison between both methods. The calculated bias between the methods (standard deviation between the difference of leg axis angle of both measurement techniques) was 2.35° and the difference was not statistically significant (M = −0.36; 95% CI = −4.97–4.25°; *p* = 0.11). There was no significant increase of systemic bias in higher varus or valgus deformities in regression analysis (ß = 0.122; CI = −0.08–0.237; *p* = 0.344). Furthermore, the leg axis analysis of all eight motion phases was inferior in accuracy to the static evaluation (Figure 4; initial contact: r_s_ = 0.684; loading response: r_s_ = 0.734; mid-stance: r_s_ = 0.806; terminal-stance: r_s_ = 0.815; pre-swing: r_s_ = 0.668; initial swing: r_s_ = 0.549; mid-swing: r_s_ = 0.662; terminal-swing: r_s_ = 0.745; *p* < 0.001). The range of systemic bias in the Bland–Altmann plot between both methods was higher compared to the static measurement, indicating a lower agreement with LSX. The highest systemic bias was detected in initial-swing (SD = 5.83; 95% CI = −14.07–8.77°) and the lowest in mid-stance (SD = 3.45; 95% CI = −7.06–6.3°).

### 3.3. Regression Analysis of Body Mass Index, Height, Body Mass, Gender, and Age

**Figure 5:**
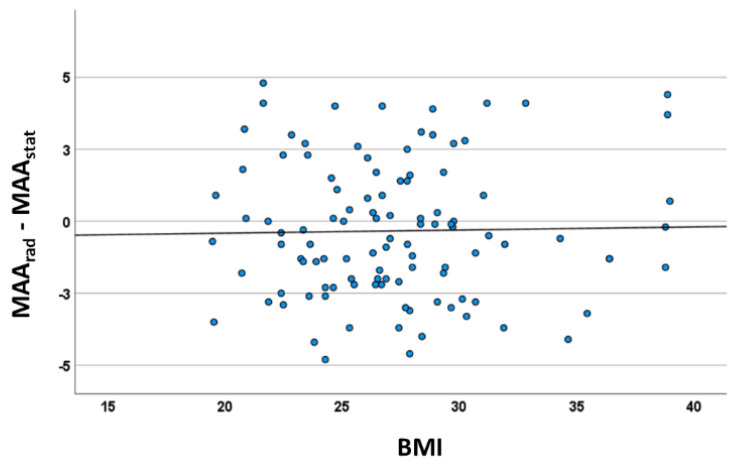
Correlation analysis between BMI and systematic bias (mean mechanical axis angle marker (MAA_stat_ vs. X-ray) indicating no influence of the BMI (r_s_ = 0.012; *p* = 0.903).

The body mass index was not associated with the difference between measurement methods (Figure 5; ß = −0.21; CI = −1.05–0.63°; *p* = 0.63)). The regression analysis of height (ß = −0.59; CI = −0.40–0.13°; *p* = 0.33), body mass (ß = 0.95; CI = 0.07–0.17°; *p* = 0.36) and gender (ß = −1.14; CI = −2.38–0.1°; *p* = 0.07) did not show any significant association with the systemic bias. Interestingly, a weak negative association could be detected for age (ß = −0.04; CI = −0.07–0.01; *p* = 0.007). The Kellgren–Lawrence score was either 0 or 1 among all subjects and did not affect any values of this outcome analysis.

## 4. Discussion

This study presents a non-invasive, radiation-free measurement technique that was used to evaluate knee joint alignment in the frontal plane in a large cohort of 102 adult patients. The most important findings of this study are that (1) there is no statistical difference between LSX and MCA in the accuracy of alignment evaluation, which is supported by the high correlation between the two methods and the agreement of the Bland– Altman plot; (2) dynamic evaluation of the leg axis shows different results when comparing to static analysis; (3) the accuracy of MCA is not influenced by BMI, height or body mass. 

Malalignment plays a major role in the development of OA and, therefore, is important to address in all therapeutic concepts of knee surgery. Consequently, an evaluation of the leg axis by X-ray is a hallmark diagnostic tool in orthopedic care. Specifically, LSX is currently performed frequently before regenerative surgery but also for planning of knee arthroplasty.

Stief et al. [15] validated a motion capture system for static alignment analysis against LSX and demonstrated a strong correlation (r_s_ = 0.808, *p* < 0.001) in 46 adolescents with idiopathic varus and valgus malalignment during temporary hemi-epiphysiodesis. In contrast, they reported a negative influence of the body mass index (BMI) on the accuracy of the technique. In their study, the bias increased from 0.7° (BMI ≤ 25) to 3.7° (BMI ≥ 25). The results of our study did not show any association of BMI influence in the accuracy of the measurement technique. However, the two studies differ regarding patient age and size of the patient cohort. Correlation analysis between different measurement techniques was also performed by Mündermann et al. [16], who demonstrated a strong correlation (r = 0.74, *p* < 0.001) of static analysis from skin markers on anatomical landmarks by a stereophotogrammetric system with radiographically measured mechanical axis angle (MAA) for patients with medial compartment knee OA [16]. Furthermore, Kornaropolus et al. [17] reported similar findings for frontal plane alignment evaluation against computer tomography (CT) in functional posture analysis by the use of skin markers before and after total knee arthroplasty (r = 0.91, *p* < 0.001). They demonstrated that other non-invasive measurement techniques (goniometer, caliper, incliniometer) were significantly inferior to the motion capture technique.

This study investigated the influence of motion on the alignment measurement. Leg axis analysis in dynamic conditions resulted in a lower agreement between measurement techniques, especially in the swing phases. In line with these results, Böhm et al. reported, in a comparison study between dynamic joint moments and static MAA from LSX, a significant correlation (r = 0.97, *p* < 0.001) in young patients treated by guided growth [18]. Hurwitz et al. reported a high correlation between dynamic joint moments and radiographic leg axis evaluation in patients with knee osteoarthritis [19]. Dynamic evaluation can be of particular importance in patients with dynamic valgus and varus malalignment. In our study, dynamic MAA was compared to the gold standard LSX. Therefore, it cannot be evaluated whether the higher differences in motion show compensation mechanisms or dynamic components or are the result of lower accuracy. Patients with a borderline degree of malalignment can profit from dynamic evaluation for decision-making for operative therapy [20]. The indication for hemi-epiphysiodesis is obtained by evaluation of LSX. In some cases, a clear recommendation for surgery cannot be given. In these cases, dynamic evaluation can further support the decision-making process [18]. On the other hand compensation mechanisms can lead to physiological leg axis of the knee joint in dynamic evaluation [21]. Wang et al. [22] reported that external foot rotation was able to compensate for adduction moments at the knee joint. Dynamic evaluation of load-bearing can also be used for evaluation of OA, which has been reported to shift in disease progression [23]. Miyazaki et al. [24] reported, in a study of 32 patients with knee osteoarthritis, a positive correlation between elevated adduction moments and disease progression. Dynamic evaluation of the load pattern was reported to be a diagnostic tool for prediction of OA progression. Hunt et al. [12] investigated, in a cross-sectional cohort study of 80 patients, a high correlation (r = 0.84) between marker-based dynamic alignment and static radiographs, but reported differences between static and dynamic conditions. Specogna et al. [25] evaluated the influence of weight-bearing on the radiographic measurement of malalignment in 40 patients with varus gonarthrosis. They determined that static and dynamic leg axis evaluation were different and peak knee adduction moment correlated moderately with the mechanical axis angle (MAA).

Radiographic evaluation can also be different between raters. Patient positioning is especially important for standardization [25]. Khare et al. [26] evaluated the accuracy of static antero-posterior radiographs for leg alignment and found that foot rotation and X-ray projection center had an influence on landmark measurement errors and estimation of leg alignment. The maximum of systemic error was 1.46° for the femoral mechanical axis and 0.66° for the tibial mechanical axis. Reliability of measuring mechanical axis alignment of long leg radiographs after total knee arthroplasty was analyzed by Bowman et al. [27]. They assessed both intra- (within different groups of professionals) and inter-observer reliability including an orthopaedic consultant, a senior orthopaedic registrar, a junior orthopaedic registrar, and a medical student. They reported a high intra-observer reliability class correlation (r > 0.9) of all professionals prior to surgery. Interestingly, the ICC was lower after total knee arthroplasty (r = 0.7 for medical students). Intra-rater reliability was high in all raters (r > 0.9). However, the authors did not analyze the inter-rater reliability between the groups of different professionals. Standard deviation of difference in mechanical axis angle was 1.3–2.3°. Similar results were also presented by Babazadeh et al. [28], with an intra-observer reliability r > 0.98; a coefficient of repeatability <1.1°; and inter-observer reliability r > 0.960 using LSX and r > 0.970 using CT, with a coefficient of repeatability <2.8° in CT scans. Moreover, weight-bearing can influence the coronal knee alignment calculation (intra-class correlation coefficient non-weight-bearing r = 0.657 vs. weight-bearing r = 0.878) [29]. Taken together, the error by patient positioning, compliance in weight bearing, and inter-observer differences impacts the accuracy of long leg radiographs. The alternative measurement by a motion capture leg axis analysis can be claimed to be comparable to static long leg radiographs and CT scans. Nevertheless, the intra- and inter-rater reliability is also an issue in the positioning of the markers on the bony landmarks. Further studies need to evaluate the accuracy of different raters.

There are specific limitations in motion capture analysis which must be considered. MCA is an indirect measurement based on the tracking of anatomical landmarks. An analysis of underlying pathologies such as knee osteoarthritis, bone lesions, or other structural deformities cannot be provided. Moreover, a precise analysis of the femoral and tibial compartment involvement, for example, for planning an osteotomy (tibial vs. femoral) is currently not possible with a motion capture technique. A combination of focused radiographs (e.g., knee in frontal and sagittal planes) and MCA for leg axis analysis could be beneficial and consequently reduce the exposition to ionizing radiation. Nevertheless, high investment, human resources, and time for a motion capture analysis of up to 30 min must be considered.

Marker-based leg alignment analysis enables static and dynamic evaluation and is often performed in a whole-body motion analysis, including the spine and pelvis. Motion capture leg axis analysis can therefore be an additional diagnostic tool for follow-up evaluation.

## 5. Conclusions

Mechanical femorotibial axis (MAA) of marker-based non-invasive static measurement strongly correlates with the MAA of long leg radiograph alignment analysis (LSX). Dynamic evaluation of the MAA increased the systemic bias compared to static measurements. The BMI did not influence the accuracy of both techniques. Marker-based alignment analysis can be an alternative to X-ray for evaluation and follow-up analysis of the frontal plane and can reduce the accumulation of radiation in individuals.

## Figures and Tables

**Table 1:** Descriptive patient characteristics (N = 102, data sets = 133).

Sex (m/f)	62/40
Age (years)	53.8 (±15.4)
Body mass (kg)	85.3 (±18.7)
Height (cm)	176.7 (±10.7)
BMI (kg/m^2^)	27.2 (±4.6)
Genu varum/valgum (data sets)	95/38
Conservative/operative (cases)	41/61

## Data Availability

Not applicable.
